# Distinct Visual Processing of Real Objects and Pictures of Those Objects in 7- to 9-month-old Infants

**DOI:** 10.3389/fpsyg.2016.00827

**Published:** 2016-06-13

**Authors:** Theresa M. Gerhard, Jody C. Culham, Gudrun Schwarzer

**Affiliations:** ^1^Department of Developmental Psychology, Faculty of Psychology and Sports Science, Justus-Liebig-University GiessenGiessen, Germany; ^2^Department of Psychology, Brain and Mind Institute, The University of Western OntarioLondon, ON, Canada; ^3^Centre for Mind/Brain Sciences, University of Trento, MattarelloItaly

**Keywords:** object processing, visual habituation, real objects, pictures, infants

## Abstract

The present study examined 7- and 9-month-old infants’ visual habituation to real objects and pictures of the same objects and their preferences between real and pictorial versions of the same objects following habituation. Different hypotheses would predict that infants may habituate faster to pictures than real objects (based on proposed theoretical links between behavioral habituation in infants and neuroimaging adaptation in adults) or to real objects vs. pictures (based on past infant electrophysiology data). Sixty-one 7-month-old infants and fifty-nine 9-month-old infants were habituated to either a real object or a picture of the same object and afterward preference tested with the habituation object paired with either the novel real object or its picture counterpart. Infants of both age groups showed basic information-processing advantages for real objects. Specifically, during the initial presentations, 9-month-old infants looked longer at stimuli in both formats than the 7-month olds but more importantly both age groups looked longer at real objects than pictures, though with repeated presentations, they habituated faster for real objects such that at the end of habituation, they looked equally at both types of stimuli. Surprisingly, even after habituation, infants preferred to look at the real objects, regardless of whether they had habituated to photos or real objects. Our findings suggest that from as early as 7-months of age, infants show strong preferences for real objects, perhaps because real objects are visually richer and/or enable the potential for genuine interactions.

## Introduction

Recent research on human object perception and recognition has increasingly questioned the ecological validity of using pictures of objects (such as photos or line drawings) as a proxy for real objects (Snow et al., 2011, 2014). After all, real objects differ from pictures, even perfectly matched photos, in many attributes including the availability of binocular depth cues (stereopsis) and motion-based depth cues (motion parallax), consistency between binocular and monocular depth cues, and the potential to act upon the objects. Here we review evidence that adults have a *real-object advantage* (that is, better performance for real objects than pictures) on a variety of tasks, that the difference between real objects and pictures may be reflected in neural processing differences, and that infants also behave differently toward real objects vs. images. Considering this background, the primary goal of the present study was to investigate 7- and 9-month-old infants’ visual habituation patterns to real objects and photorealistic pictures of the same objects as well as their preferences for the same items presented in real and picture format following habituation.

### Visual Perception of Real Objects and Pictures in Adults

In patients with visual form agnosia, object recognition performance is often enhanced with respect to real objects relative to pictures; a phenomena termed the real-object advantage (Riddoch and Humphreys, 1987; Young and Ellis, 1989; Servos et al., 1993; Humphrey et al., 1994; Chainay and Humphreys, 2001). Additional three-dimensional (3D) object information provided by binocular depth cues (including cues to actual object size based on perceived distance) and richer surface properties such as color, and texture are assumed to contribute to this effect (Servos et al., 1993; Chainay and Humphreys, 2001). More recent research has also shown behavioral advantages for real objects in neurologically intact research participants. Bushong et al. (2010), for example, found that participants in a neuroeconomics study were willing to pay about 50% more when bidding on items (food or trinkets) presented as real objects vs. photographs or text labels. Interestingly, however, they also found that placing a large transparent (Plexiglas) barrier between the participants and stimuli eliminated the effect, suggesting that valuation was not driven by low-level visual features such as binocular disparity, which did not change with the barrier, but rather by the accessibility of the food. Moreover, Snow et al. (2014) demonstrated a differential effect of stimulus format on episodic memory performance. In an initial encoding phase subjects were asked to memorize a total of 44 common household items that were presented either as real objects, color photographs, or black and white line drawings. Following stimulus encoding all subjects were tested for free recall and recognition performance. Results showed that for both episodic memory measures subjects’ performance was superior for real objects compared to color photographs and line drawings.

### Neural Processing of Real Objects and Pictures in Adults

Recent research has raised the possibility that real objects not only evoke different behavior but may also invoke differences in neural processing. Most notably, Snow et al. (2011) used fMRI to investigate whether real objects and photos evoked similar levels of blood-oxygen-level-dependent (BOLD) activation and whether the response decreased with repetition. Repetition attenuation (also called fMRI adaptation or priming) for images has been commonly observed in object-selective areas; specifically, the presentation of a repeated image (e.g., duck-duck or baseball-baseball) evokes less activation than the presentation of different images (e.g., duck-baseball; Grill-Spector et al., 1999). Such effects are thought to reflect weaker, faster or more finely tuned neural processing for stimuli that have been previously processed, though the exact mechanisms are debated (Grill-Spector et al., 2006). Snow et al. (2014) measured fMRI activation while participants simply viewed pairs of repeated or unrepeated stimuli that were presented either as real objects or visually matched photographs. As expected from past research, robust repetition effects were found for trials containing repetitions of object pictures throughout a wide variety of object-selective brain regions. Surprisingly, however, similar effects were rather weak, if not entirely absent, on trials involving real objects. Notably, the differences in repetition effects were observed even though overall response levels were comparable for objects and photos. These results suggest that the neural processing of real objects differs from photos. One possible interpretation may be that real objects continue to be processed longer than images, perhaps related to the behavioral findings that real objects are more highly valued (Bushong et al., 2010) and memorable (Snow et al., 2014). The fundamental reason for the differences between real objects and images is yet to be determined, but may include differences in stereoscopic depth cues, consistency of monocular and binocular cues to object shape, and the tangibility and potential for actions provided by real objects.

### Infants’ Visual Perception of Real Objects and Pictures

Behavior and neural processing is enhanced not only in adults but also in infants when they process real objects compared to pictures. Between 5- and 7-months of age infants have developed sufficient visual abilities to discriminate real objects from pictures (Rose, 1977; DeLoache et al., 1979; Slater et al., 1984; Kavšek et al., 2012) but also to perceive their similarities (e.g., Jowkar-Baniani and Schmuckler, 2011). Together with studies that examined infants’ manual exploration behavior (DeLoache et al., 1998; Pierroutsakos and DeLoache, 2003; Yonas et al., 2005; Ziemer et al., 2012), these studies provide first indications for a cognitive distinction and thereby for a distinct processing of real objects and pictures.

Infants neural processing also appears to be faster for real objects. In an event-related potentials (ERP) study, Carver et al. (2006) explored the temporal correlates of visual object recognition in 18-month-old infants. One group of infants saw either familiar or unfamiliar real toys, whereas the other group saw pictures of either familiar or unfamiliar toys. Although differences between familiar and unfamiliar toys were seen in late ERP components for both real objects and pictures, differences in early ERP components were found only for the real objects, suggesting that real objects are processed faster than pictures.

Real objects may also be remembered better than pictures in infants, consistent with findings from adults (e.g., Snow et al., 2014). For example, Rose et al. (1983) revealed that 12-month-olds’ recognition memory for real objects is less dependent on task specifics such as encoding time. They investigated infants’ intramodal and crossmodal transfer from real objects to their pictorial representations. On three trials infants were first visually (intramodal group) or tactilely (crossmodal group) familiarized with real objects, and afterward tested for visual object recognition with the real objects and their pictorial representations. In a first experiment with a 30-s familiarization period, infants in the intramodal group showed substantial object recognition for both real objects and pictures, whereas infants in the crossmodal group revealed significant recognition only for real objects. However, when familiarization time was reduced to 15 s in the intramodal group, infants still recognized real objects but no longer their pictorial representations. Additionally, Ruff et al. (1976) examined 3- and 5-month-old infants’ speed in learning to recognize unfamiliar real household objects vs. color photographs of those objects. They created a task that tested infants’ visual recognition memory at different points in the experiment and, therefore, verified whether recognition would appear faster for real objects or pictures. Each session involved six familiarization trials with an identical picture or real object interspersed with two paired-comparisons of the familiarization object and a novel object to test for visual object recognition. Their main finding was that only the 5-month-olds exposed to real objects showed solid recognition memory after half of the familiarization trials, indicated by robust novelty preferences from trial three on. Five-month-old infants that were exposed to color photographs, instead, showed no signs of recognizing the photographs throughout the session. Hence, when familiarized to a real object infants seemed to be able to create a mental representation of that object but not when they were familiarized to pictures. The authors concluded that from 5-months on infants learn to recognize real objects faster than pictures of objects. In addition, in the 5-months-olds overall attention to real objects, relative to pictures, declined significantly during familiarization indicated by a larger decrease in fixation time from the first familiarization trial to the last familiarization trial.

However, it is possible that these differences in infants’ recognition performance as well as in their familiarization to real objects vs. pictures obtained by Ruff et al. (1976) arose from an insufficient ability to properly perceive pictorial depth cues within the photographs of the complex and unfamiliar household objects they used as stimuli. This is relevant since the perception of depth cues in pictures is a crucial requirement for processing pictures in a similar way as corresponding objects. As a matter of fact, studies that tried to establish the age in which infants start to respond to pictorial depth cues provided divergent results (Kavšek et al., 2012). While preferential reaching methods determine the time of infants’ sensitivity to pictorial depth cues between 5 and 7 months of age, research using looking-time methods (habituation-dishabituation and preferential-looking studies) arrive at an age of about 3 to 6 months, largely depending on whether they controlled for an influence of low-level stimulus features on infants’ experimental performance. In this case, responsiveness to pictorial depth cues unambiguously emerged only with about 6 months (for a review see Kavšek et al., 2012).

### Linking Neural and Infant Habituation Effects

Intriguingly, Turk-Browne et al. (2008) have suggested possible theoretical links between the effects of repetition effects in adult neuroimaging studies and habituation effects in infant behavior studies. Specifically, both approaches typically report decreased responses resulting from stimulus repetition (though increased responses can also occur). These effects can be used to explore representations by examining whether the repetition effects are sensitive to changes to specific attributes of the repeated stimuli. Moreover, they suggest that both approaches may afford increased sensitivity compared to alternative approaches; that is, fMRI repetition effects can reveal effects absent in simple contrasts of activation levels (as observed in the Snow et al., 2011 data) and looking times may reveal earlier sensitivity to certain stimulus features than methods based on measuring actions like reaching or grasping which develop later than vision.

Although there may be some analogies between the techniques, there are also numerous reasons to think that infant habituation and adult fMRI repetition effects are not directly comparable. Most obviously, the participants’ ages are very different. In addition, both infant habituation and adult fMRI repetition effects could arise from a wide variety of factors, including memory (Henson, 2003), attention (Moore et al., 2013), processing speed (James et al., 2000), or predictability (Summerfield and de Lange, 2014). fMRI repetition effects can differ between brain areas and some effects are consistent with behavioral signatures of repetition while others are not (e.g., Xu et al., 2007).

Our research question provides an opportunity to conduct a comparison between adult fMRI repetition effects (Snow et al., 2011) and infant habituation results, as shown here using a similar paradigm. Specifically, in both studies we can examine the effects of repeating presentations of real objects or pictures. If Turk-Browne et al. (2008) are correct in surmising an analogy between the approaches, we might expect similar effects in the two types of data; otherwise, we might expect that the specific factors contributing to the two types of effects may lead to inconsistencies in the results.

### The Current Study and Hypotheses

Here we examined whether and to which extent infants in their 1st year of life show distinct visual habituation to real objects vs. pictures of the same objects. In multiple trials, we presented 7- and 9-month-old infants with either a real toy or a realistic picture of that toy. In a subsequent test phase, infants’ visual recognition memory regarding the objects was evaluated by presenting pairs of the habituation object together with its counterpart in the other format. Note that our test period differs from past work (e.g., Ruff et al., 1976) in comparing two formats – real and picture – of the same object, rather than comparing two different objects in the same format.

Several alternative outcomes are possible and would support different theories. First, different outcomes are possible for the habituation phase. Given the proposed theoretical relationship between neural and infant habituation effects (Turk-Browne et al., 2008), the findings of robust repetition effects for pictures but not real objects in adult fMRI experiments (Snow et al., 2011) would predict robust infant habituation effects for pictures but little or no habituation for real objects (combined with little difference in overall looking times as no differences in overall fMRI activation were observed between real objects and pictures in the fMRI). As one alternative hypothesis, if infants find real objects more engaging because of the richer information they provide (including binocular depth and motion parallax) and their potential for interaction, this would predict longer looking times for real objects. As another alternative hypothesis, if infants are struggling to process pictures due to the relative unfamiliarity of pictures compared to real objects and to the conflicting cues to depth that arise with pictures, this would predict longer looking times for pictures.

Second, different outcomes are possible for the test phase. Assuming infants are able to discriminate a real object from its photo counterpart, they are expected to show preferential looking toward one of the test items. Based on novelty, the prediction would be a preference for the previously unseen stimulus, that is, the real object following adaptation to its photo counterpart and the photo following adaptation to its real counterpart. Based on violation of expectations, the prediction would be a preference for the photo object, which violates the normal relationship between binocular and monocular depth cues, regardless of habituation format. Finally, based on how engaging and valuable the stimulus is, the prediction would be a general preference for the real object, which affords real interaction, regardless of habituation format. DeLoache et al. (2003) argue that even though on a visual level young infants can already discriminate between actual objects and pictures of objects, the full understanding of the representational nature of pictures seems yet to be obtained with 9 months of age (see also Yonas et al., 2005; Ziemer et al., 2012). From this it could be inferred that infants take pictures for objects and would show no clear preference.

For both the habituation phase and the test phase, different theories can yield different, even opposite outcomes. If a clear outcome is obtained, this suggests that one theory yields better predictions that the others, though it is possible effect sizes may be tempered by several factors.

## Materials and Methods

### Ethics Statement

The present study has been realized in accordance to the German Psychological Society (DGPs) Research Ethics Guidelines. For each infant, written consent for participating in the study was obtained from the parents.

### Participants

The final sample consisted of 61 healthy and full-term 7-month-old infants at the mean age of 7 months 17 days (*SD* = 7 days; 28 girls and 33 boys) and 59 nine-month-old infants at the mean age of 9 months 19 days (*SD* = 8 days; 28 girls and 31 boys). The data from additional 13 seven-month-old and 8 nine-month-old infants were excluded from the final sample due to fussiness (19), experimenter error (1), or failure of the technical equipment (1). Infants were recruited by obtaining their birth records from local municipal councils and neighboring communities. Participants were predominantly Caucasian infants who lived in Giessen and suburban areas of Giessen.

### Stimuli

Stimuli consisted of four different, aged-based toys (mouse, car, frog, and bear) and photographs that were as realistic as possible. The width of the objects ranged from 10.0 to 13.5 cm and the height from 8.5 to 14.0 cm.

**FIGURE 1:**
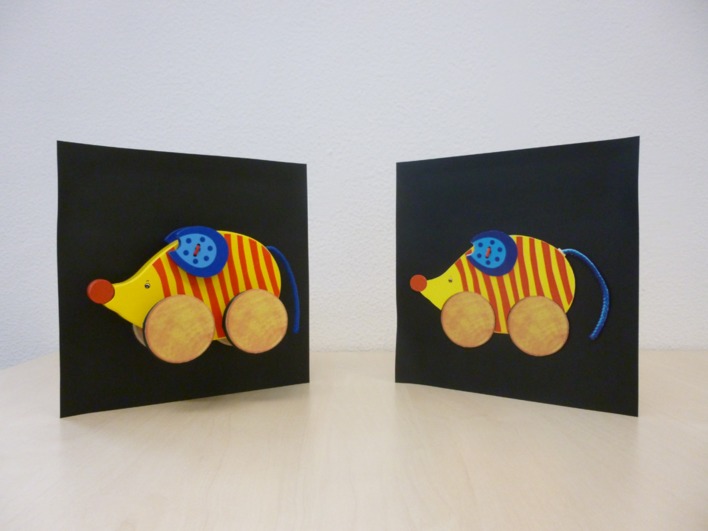
**Example of a stimulus pair.** Stimuli a slightly tilted inward to get a better view on the real object (left hand side) and its matched photograph (right hand side).

Photographs were taken with a good-quality digital camera (Sony DSC-W170 digital camera, 10.1-megapixel resolution). All pictures were taken in the cabin of the experimental setup with the camera placed at the infant’s point of view and such that the viewpoint and lighting was the same as that for the real object. Photos were adjusted to real objects pertaining to contrast and brightness with Adobe Photoshop CS6 and printed on photo paper such that the physical size of each matched that of the corresponding real object. For the purpose of stimulus presentation, both real objects and their photographs were fixed to a cardboard which was laminated with black polypropylene and fixed to a wooden box. The final stimulus set consisted of eight stimuli divided into four pairs of real objects and their matched photographs (**Figure 1**).

### Apparatus

The experiment was conducted in a white rectangular cabin with an open front to accommodate a caregiver and her child. The child was seated on the caregiver’s lap at a distance of approximately 60 cm from the stimuli beyond the infants’ reach. From the rear wall of the cabin a 42.5 × 32 cm-sized window was cut out which could be opened and closed via a sliding door made of two black pieces of cardboard. By opening the sliding door a 51 cm × 33 cm × 39 cm enclosed stage appeared which served for presenting the stimuli. For the purpose of placing the stimuli onto the stage its top side was open. The floor of the stage was made of dark chipboard with markers for the correct positioning of the stimuli during the experiment.

During testing, one experimenter measured infants’ fixation times while a second experimenter presented the stimuli. Both experimenters were located behind the setup and hidden from view. The entire session was recorded on a VCR using a low-light video camera which was attached to a peephole in the back of the cabin 5.5 cm above the sliding door. Connected to the camera was a television screen from which infants’ gaze behavior could be observed by the first experimenter. Fixation time measurements were taken via a Fujitsu Siemens Lifebook running BABY, a computer software for conducting habituation and preferential looking time experiments (Krist, 2001).

### Procedure

All infants were tested in individual sessions. To prevent parents from influencing their babies’ fixation times they were asked to keep their eyes closed and to refrain from talking for the duration of the experiment.

To test infants’ visual processing and discrimination of real objects vs. pictures, a visual discrimination task was conducted which consisted of a habituation phase and a test phase. In the habituation phase, infants were exposed to one of the four toys either as a real object or picture. The number of infants administered to the four toys in the two different formats (real object and picture) was counterbalanced. To attract infants’ attention each trial began with the ringing of a bell from behind the stimuli. After opening the sliding door the habituation stimulus became visible in the middle of the stage. As soon as infants began fixating the stimulus the first experimenter started measuring fixation times by pressing a button. Fixation durations under 1 s were not counted as fixating the stimulus. Trial length was based on infant’s fixation of the display. Each trial ended either 2 s after the infant turned her gaze away from the stimulus or after 60 s had passed. The trial continued if the infant returned her attention to the habituation stimulus during the 2-s interval. At the end of a trial the sliding door was closed and the procedure of stimulus presentation described above was repeated. The habituation phase ended when the average fixation time to the stimulus within the last three habituation trials declined to 50% of the average time within the first three habituation trials or when a maximum of 14 habituation trials had been presented. Altogether, infants saw a minimum of 6 and a maximum of 14 habituation trials.

The test phase included three trials with paired-comparisons of the habituation stimulus (real object or picture) together with its counterpart in the other format (novel stimulus). In the first test trial, the novel stimulus was positioned on the left and the habituation stimulus on the right side of infants’ gaze direction. After each trial stimuli positions were interchanged. Note that during the habituation phase, only a single item was presented at a time (a given toy either in real or picture format); whereas during the test phase, two items (the same object presented in real and picture format) were presented. An approximately 14-cm distance between the edges of the stimuli ensured reliable measurements of whether infants fixated to the left or to the right test stimulus. Following the general procedure of stimulus presentation from the habituation phase, fixation time measurements started as soon as infants attended to one out of the two stimuli on the stage. Depending on the experimenter’s perspective fixations to the right or left test stimulus were indicated by right or left button presses. As in the habituation phase, fixation durations under 1 s were not counted as fixating the stimuli and trial length was again based on infant’s fixation of the display. Hence, each trial ended either 2 s after the infant turned her gaze away from the stimuli or after 60 s had passed. The trial continued if the infant returned her attention to one out of the two test stimuli during the 2-s interval.

Trained observers who were naïve to the hypotheses under investigation recorded the time infants spent fixating on the stimuli using videotapes of the sessions. The inter-observer reliabilities of habituation and test phases for both age groups exceeded 0.9.

## Results

Experimental results were divided into habituation and test phases.

### Habituation Phase

Habituation phase analyses were performed based on Singh et al. (2015) approach, which quantified fixation times for the first two and last two habituation trials. All 120 participants (61 seven-month-olds and 59 nine-month-olds) were included in the analyses of the habituation phase. Fifty-eight of the infants were habituated to real objects (29 seven-month-olds and 29 nine-month-olds) and 62 were habituated to pictures (32 seven-month-olds and 30 nine-month-olds). We conducted a 2 × 2 × 2 repeated-measures ANOVA to examine infants’ looking times with habituation trial number (first two habituation trials and last two habituation trials) as a within-participants variable and age group (7-month-olds or 9-month-olds) and habituation stimulus format (real object or picture) as between-participants variables. A preliminary ANOVA with a fourth factor of object identity (mouse, car, frog or bear) revealed no significant main effect of object identity nor interactions with object identity (all *F*s < 1.38, all *p*s > 0.25); thus, we collapsed across this factor to simplify the analyses.

**FIGURE 2:**
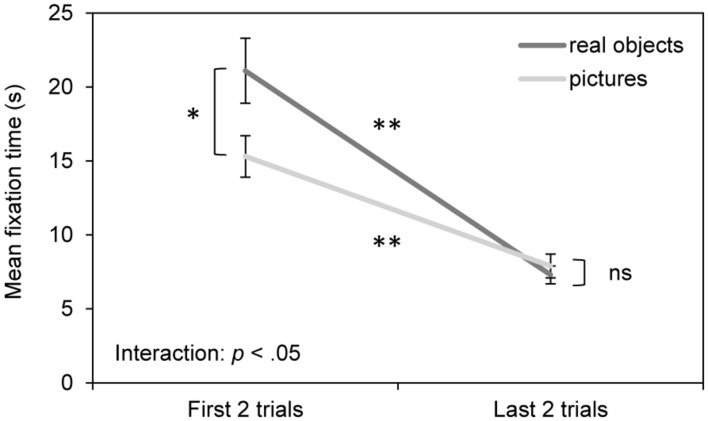
**Results of the habituation phase.** Mean fixation time (s) for real objects and pictures during the first two habituation trials and the last two habituation trials. Error bars indicate the standard error of the mean. *p < 0.05, **p < 0.001.

Most interestingly, as shown in **Figure 2**, infants spent significantly more time looking at stimuli in the first two trials compared to the last two trials and this effect was significantly more pronounced for real objects than pictures. That is, infants spent more time looking at real objects than pictures initially; however, over the course of habituation, the looking times for real objects dropped at a faster rate than for pictures until they were similar between the two formats.

Statistically, this pattern is indicated by the 3-way ANOVA, which revealed both a main effect of habituation trial number, *F*(1,116) = 72.15, *p* < 0.001, $\eta_{p}^{2}$ = 0.383, and an interaction between habituation trial number and habituation stimulus format, *F*(1,116) = 6.52, *p* < 0.05, $\eta_{p}^{2}$ = 0.053. *Post hoc t*-tests revealed significant decrements in looking times with habituation for both stimulus formats, *t*(57)_real objects_ = 6.36, *p* < 0.001, *d* = 1.09, and, *t*(61)_pictures_ = 5.37, *p* < 0.001, *d* = 0.84, and significantly longer looking times for real objects on the first two trials, *t*(118) = 2.24, *p* < 0.05, *d* = 0.41, but not the last two trials, *t*(118) = -0.65, *p* > 0.05, *d* = -0.12. In addition, the ANOVA revealed a main effect of age, such that 9-month-old infants fixated longer on the stimuli than 7-month-old infants; however, there was only a trend toward an interaction between age group and habituation trial number, *F*(1,116) = 3.53, *p* = 0.063, $\eta_{p}^{2}$ = 0.030 and no significant three-way interaction of age group × habituation trial number × habituation stimulus format, *F*(1,116) = 2.44, *p* > 0.05, $\eta_{p}^{2}$ = 0.021. In addition, there was a trend toward a main effect of format but this must be considered in light of its interaction with habituation trial number.

We also analyzed infants’ accumulated looking times (that is the sum of looking times across all trials in the habituation phase) via a 2 × 2 ANOVA with age group and habituation stimulus format as between-participants variables. Again, a preliminary ANOVA with object identity as a third factor yielded no significant main effect of object identity nor interactions with object identity (all *F*s < 1.62, all *p*s > 0.19); thus, we collapsed across this factor to simplify the analysis.

Concerning accumulated looking times, there was a main effect of age group, *F*(1,116) = 4.24, *p* < 0.05, $\eta_{p}^{2}$ = 0.035, but more importantly no main effect of habituation stimulus format nor an interaction between the two factors (all *F*s < 0.71, all *p*s > 0.40). Overall, 9-month-old infants fixated longer on the stimuli than 7-month-old infants, but accumulated looking times did not differ between real objects and pictures.

### Test Phase

Prior to test phase analyses, 18 seven-month-old and 15 nine-month-old infants were excluded because they failed to reach the habituation criterion within the 14-trial maximum of the habituation phase. The data of additional 10 seven-month-olds and 4 nine-month-olds were excluded because they failed at least once on fixating to one out of the two test stimuli during the three test trials. Thus, test results are based on the data of 73 infants. Forty-one of the infants were habituated to real objects (18 seven-month-olds and 23 nine-month-olds) and thirty-two of the infants were habituated to pictures (15 seven-month-olds and 17 nine-month-olds). In order to test for infants’ visual preferences during the test phase following habituation, a preference score on the percentage of time each infant spent fixating to the novel object (real object or picture) across all three test trials was calculated by dividing fixation time to the novel object by overall fixation time multiplied by 100.

We conducted a 2 × 2 ANOVA examining the effects of the two age groups (7-month-olds or 9-month-olds) and habituation stimulus format (real object or picture) on the preference score for novel objects. A preliminary ANOVA with a third factor of object identity (mouse, car, frog, or bear) revealed no significant main effect of object identity nor interactions with object identity (all *F*s < 2.04, all *p*s > 0.11); thus, we collapsed across this factor for the following analyses.

**FIGURE 3:**
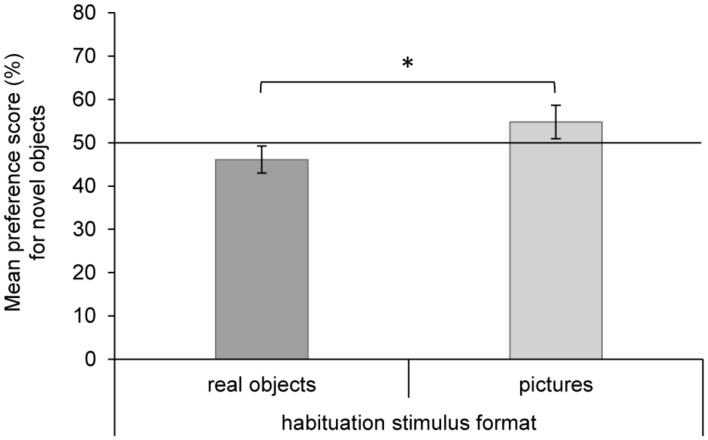
**Results of the test phase.** Mean preference score (%) for novel objects (real object or picture) in the two habituation stimulus format groups. Error bars for the preference scores are based on the 97.5% confidence intervals, which indicate whether or not the average preference (Bonferroni corrected) was significantly greater or lower than 50%. *p < 0.001.

The 2 × 2 ANOVA on the preference score for novel objects with age group and habituation stimulus format as between-participants variables revealed a significant main effect of habituation stimulus format, *F*(1,69) = 17.38, *p* < 0.001, $\eta_{p}^{2}$ = 0.201, but no additional main effect of age group or interaction (all *F*s < 0.85, all *p*s > 0.36). Infants who were habituated to real objects showed a familiarity preference (*M* = 46.1%, *SE* = 1.3), indicating that they kept preferring to look at real objects during the test. For infants who were habituated to pictures of objects, our analyses revealed a preference for novel objects (*M* = 54.8%, *SE* = 1.6) and therefore, again, for real objects (**Figure 3**). In order to contrast the preference scores for novel objects separately for the two habituation stimulus formats against chance level, *post hoc* single *t*-tests were performed (Bonferroni corrected). The *t*-tests confirmed the preference for real objects to be significantly different from chance level for infants who were habituated to real objects, *t*(40) = -2.93, *p* < 0.01, *d* = 0.46, as well as for infants who were habituated to pictures of those objects, *t*(31) = 2.95, *p* < 0.01, *d* = 0.52.

## Discussion

The principal motivation of the present study was to examine 7- and 9-month-old infants’ visual habituation to real objects and pictures of those objects to provide new insight into the basic visual processing of objects varying in format. Our results revealed three key findings: (1) infants spent more time looking at real objects than pictures during the initial habituation trials; (2) they habituated to real objects faster than to pictures such that, at the end of habituation, they looked equally at the stimuli regardless of format; and (3) following habituation, during test trials where a habituated stimulus was paired with the same stimulus in the other format, infants preferred looking at the real object, regardless of whether they had become habituated to the real object or picture version. These effects did not differ significantly between the two age groups. Moreover, differences in the habituation and the test phase were not determined by differences in accumulated looking times during habituation, which was the same for real objects and pictures, although the older infants did spend more time fixating on the stimuli overall (including during the initial presentations). At first sight, these more pronounced fixation times in the older infants may seem unusual because traditional habituation research often finds shorter fixation durations with age (for an overview see Colombo and Mitchell, 2009); however, the relationship between age and fixation duration in infant attention may not be straightforward and may depend on the type of stimuli employed. For complex and interactive stimuli, fixation duration seems to increase with age (Courage et al., 2006; Reynolds, 2015). Because we presented highly relevant age-based toys within a live setup, older infants may have been particularly engaged by the stimuli, leading to greater fixation times overall (including at the beginning of habituation when infants’ baseline attention was assessed).

Our habituation data reveal that children demonstrate a real-object advantage as previously demonstrated in adults (Riddoch and Humphreys, 1987; Servos et al., 1993; Humphrey et al., 1994; Chainay and Humphreys, 2001; Bushong et al., 2010; Snow et al., 2014) and children from infancy on (Ruff et al., 1976; Rose et al., 1983). Moreover, they are in agreement with electroencephalography results that suggested enhanced processing of real objects compared to pictures in infants 18-months of age (Carver et al., 2006), and suggest that the real-object advantage extends to infants as young as 7-months-old.

A second aim of the present study was to examine infants’ ability to discriminate real objects from pictures of objects. The present results showed that 7- and 9-month-old infants were able to discriminate real objects from pictures and that both age groups preferred to look at real objects, independent of whether they were habituated to real objects or pictures. These results are consistent with a small number of studies that have reported preferences for real objects over pictures in the absence of habituation. DeLoache et al. (1979) found that 5-month-old infants spontaneously preferred to look at real dolls than color photographs of the same dolls. In contrast, Slater et al. (1984) found a spontaneous preference for real objects in one experiment and a preference for pictures of objects in another one. However, Fantz and Nevis (1967) point to a shift with age in preference from pictures to real objects in infants which might be due to an increasing awareness of the affordances of real objects.

What is particularly striking about the present results is that the real-object preference persists even after infants have fully habituated to real objects. This aspect of the findings is not consistent with a preference for novel objects nor with a preference for items that violate expectations. Rather, it shows that real objects are more attention-grabbing even when they are familiar. This could be due to the richness of visual information provided by real objects but not pictures, including stereo depth and motion parallax, or to the fact that real objects are more compelling and valuable because they are tangible and afford actions. Certainly, the latter goes well together with a nativist claim of innate predilections that dispose the newborn infant to focus attention on stimuli that will later on have adaptive significance (Fantz, 1961), such as preferences for human speech-sounds (e.g., Vouloumanos and Werker, 2007; Shultz and Vouloumanos, 2010) or human faces (Mondloch et al., 1999).

Note that the real stimuli we employed were quite flat and shallow, so if stereo depth is a key factor, then the effects may be expected to be even larger with stimuli that have more depth structure. Future studies could tease apart the contributions of these factors by having infants view the stimuli monocularly to eliminate stereo vision, restricting head movements or employing a virtual display that keeps the view constant with head movements to restrict motion parallax, and examining groups with different degrees of hands-on vs. visual experience with the real objects.

Our results call into question a straightforward relationship between infant habituation and fMRI repetition effects, as has been proposed by Turk-Browne et al. (2008). Specifically, fMRI studies found repetition effects for pictures but not real objects (Snow et al., 2011), which would lead to a prediction that infants would also habituate to pictures but not real objects. In fact, we found the converse – greater habituation to real objects than to pictures. Despite the absence of a direct mapping of results between the two techniques, the fMRI and infant habituation studies may nevertheless reveal commonalities of a real-object advantage across the age groups and methods. The similarity lies in the finding that for “both babies and brains,” real objects are more engaging both perceptually and neurally and evoke longer processing. In fMRI, this is reflected by prolonged processing of real objects (that is, weak or absent repetition effects); whereas, in infant behavior, it is reflected by prolonged looking times. Thus, while there is merit to the proposal that infant habituation and fMRI adaptation may tap into related mechanisms (Turk-Browne et al., 2008), there also appear to be important differences in cognitive processing between infants and adults and between what is measured by behavior and fMRI. Most notably, fMRI repetition effects may result from a variety of neural mechanisms (Grill-Spector et al., 2006) and be influenced by memory (Henson, 2003), attention (Moore et al., 2013), or expectations (Summerfield and de Lange, 2014). Moreover, fMRI repetition effects are not always consistent with behavioral differences (e.g., Xu et al., 2007).

In summary, our findings indicate that 7- and 9-month-old infants show a robust preference for looking at real objects instead of their pictorial representations but upon the initial encounter and following prolonged viewing.

## Author Contributions

JC and GS conceptualized and designed the work and were involved in interpreting the data. They gave their final approval of the work to be published as well as their agreement to be accountable for all aspects of the work. JC was involved in drafting the work and in revising it critically. GS was involved in revising the work critically. TG was responsible for the acquisition, analysis, and interpretation of the data and drafting the work. She gave her final approval of the work to be published and agrees to be accountable for all aspects of the work.

## Conflict of Interest Statement

The authors declare that the research was conducted in the absence of any commercial or financial relationships that could be construed as a potential conflict of interest.
